# A Feasibility Study of Virtual Reality and 360° Video Training for Anterior Nasal Packing

**DOI:** 10.1002/lio2.70243

**Published:** 2025-09-09

**Authors:** Holly Mould, Andrew Kinshuck, Jonathan R. Abbas, Nick Culley, Elena Karakashevska

**Affiliations:** ^1^ Aintree University Hospital Liverpool University Hospitals Trust Liverpool UK; ^2^ ExR Education ExR Solutions Ltd London UK; ^3^ University of Manchester Manchester UK; ^4^ Department of Psychology University College London London UK

**Keywords:** epistaxis, simulation, virtual reality

## Abstract

**Objectives:**

Virtual reality (VR) simulators have been well established in ear, nose, and throat (ENT) training for many years. However, most are aimed at specialist trainee level or above, with no current VR ENT training packages for foundation year doctors, who perform a significant amount of out‐of‐hours ENT care. Novel VR and 360° video packages for the training of anterior nasal packing were developed and evaluated.

**Methods:**

In this feasibility study, 16 foundation year doctors were pseudorandomised to a three‐arm study: using solely the VR simulation, solely the 360° video, or the video followed by the VR simulation. Pre‐ and post‐intervention questionnaires were devised using expert consensus, and confidence, competence, objective knowledge, usability, face and content validity, and qualitative data were all evaluated.

**Results:**

There was a significant increase between pre‐ and post‐intervention confidence, competence, and knowledge for the whole cohort. The 360° video used alongside the VR simulation and alone was deemed user‐friendly. All packages achieved content validity, whilst the VR simulation did not achieve face validity, with responses indicating that the haptic feedback requires improvement. Participants found the packages engaging and immersive, encouraging safe practice in an enjoyable manner.

**Conclusion:**

These novel VR and 360° videos are engaging ways of effectively increasing confidence, competence, and knowledge in anterior nasal packing. Further adjustments should be made to the haptic feedback of the VR simulator to make it more realistic.

**Level of Evidence:**

N/A.

## Graphical abstract

The authors developed and evaluated novel virtual reality and 360° video training packages for anterior nasal packing. These resources were demonstrated to increase subjective procedural confidence and competence and objective procedural knowledge. They were viewed as an enjoyable and engaging training modality and show promise as safe and effective training tools.

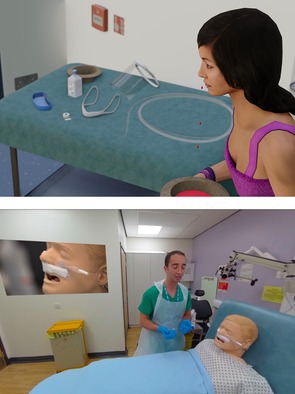

## 1 Introduction

Virtual reality (VR) is becoming an increasingly popular educational modality in medicine [1]. Numerous “VR surgical simulators” have been in use for decades as part of ear, nose and throat (ENT) surgical training [2, 3, 4, 5, 6]. The simulators are predominantly aimed at specialist trainees and have been developed for temporal bone surgery [2], endoscopic sinus surgery [3, 6] myringotomy [4], laryngoscopy and bronchoscopy [5, 7]. These simulators are effective in teaching surgical procedures [2, 6], increasing surgical confidence [8] and allowing trainees to practice in their own time with no risk of patient harm [9].

VR headsets such as the Meta Quest series (Meta Platforms, San Francisco, CA) have been developed for gaming [10]. They create a completely immersive simulated world, using a headset to create a 360° field of immersion, rather than a three‐dimensional (3D) image on a computer screen [11, 12]. Users can move and navigate within this virtual world and use handsets to interact with objects [13]. 360° videos are filmed using specialist equipment that allows the viewer to then be immersed within the video contents [14]. Despite VR simulators forming a part of ENT surgical training, very few simulators have been developed for use with VR headsets [15, 16]. Aussedat et al. developed an audiology clinic training package, and ENT residents found it immersive and realistic, and stated that it improved their knowledge and confidence in audiology [16]. Richards et al. developed a functional endoscopic sinus surgical simulator using 3D printed controllers in a mixed reality approach to surgical simulation, finding this to be a cost‐effective training package with face and construct validity [15].

Whilst specialists provide much of the in‐hours care to ENT patients in hospital, much of the out‐of‐hours care is provided by junior doctors with limited ENT experience [17]. The average undergraduate exposure to ENT in the UK is just 8.1 days [18] and during this period, students will prioritize achieving mandatory course requirements alongside learning theory; therefore, neglecting the practicalities of acute ENT management. A small number of courses delivered via mannequin simulation and lecture‐based methods have been developed to address this problem and have demonstrated an increase in participant confidence and competence in dealing with ENT emergencies [18, 19, 20]. However, as far as the authors are aware, no VR simulation packages have been developed for Foundation Year ENT training, and no studies have combined VR simulation alongside a 360° video as an educational approach.

## 2 Materials and Methods

### 2.1 Development of the VR Simulation

A VR anterior nasal packing (ANP) simulation was designed and developed by ExR Solutions Ltd. (https://exr.education/) using Unity 2018 software (Unity Technologies, San Francisco, CA) in coding language C#. A simulated assessment room in an Emergency Department with a patient with epistaxis was built and is accessible through the ExR Education application and website (exr.education) alongside the 360° video (see online Video 1 for a demonstration of both).

**VIDEO 1:** Video displaying live screen capture of the 360° video and VR simulation experiences developed for this study. Video content can be viewed at https://onlinelibrary.wiley.com/doi/10.1002/lio2.70243.

**FIGURE 1:**
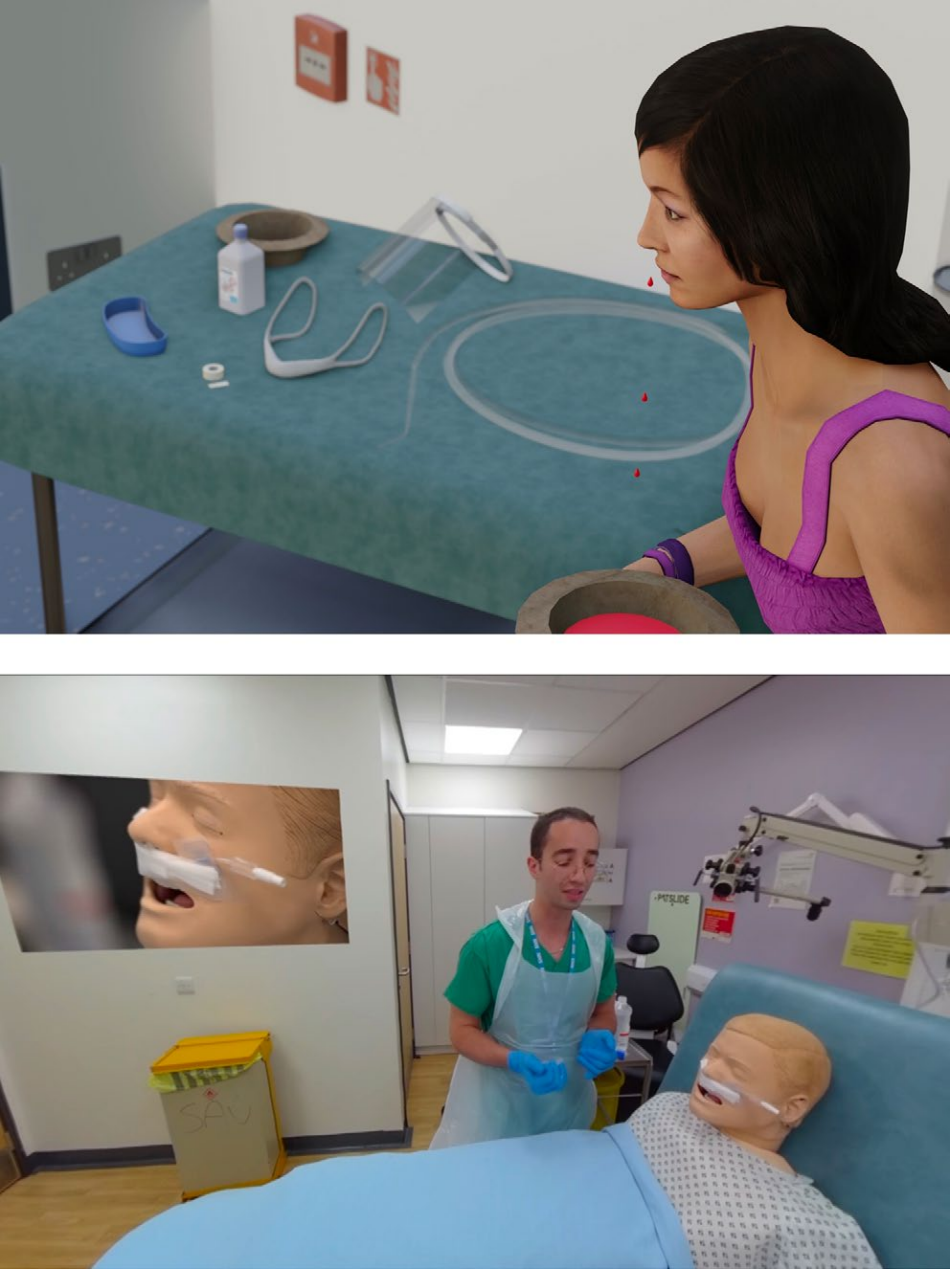
Screenshots from the VR simulation (top panel) and 360° video (bottom panel).

A script for the scenario was devised and validated with expert consensus (an ENT consultant and registrar). Using VR handheld controllers, users interact with 3D models of medical equipment in the virtual environment to prepare and perform the ANP procedure. Figure 1 displays screenshots from both the VR simulation and 360° video.

### 2.2 Development of the 360° Video

A 360° video was filmed in a surgical procedure room of an ENT tertiary center in England. The VR script was adapted for the purpose of the video and performed in simulation on a mannequin by an ENT doctor. The 360° view was augmented with close‐up cinematography of the procedural detail.

### 2.3 Subjects

Sixteen foundation year doctors (those in the first 2 years post‐graduation) from a North West deanery were recruited and participants were pseudorandomised in order to maintain equal numbers in each of the three study arms: VR simulation, 360° video, or VR simulation followed by the 360° video (combined).

### 2.4 Ethics

This study was approved as a service evaluation by the Trust's clinical audit department. Participation was voluntary. A digital consent form was signed by each participant, and a participant information sheet was supplied to them.

### 2.5 Intervention

**FIGURE 2:**
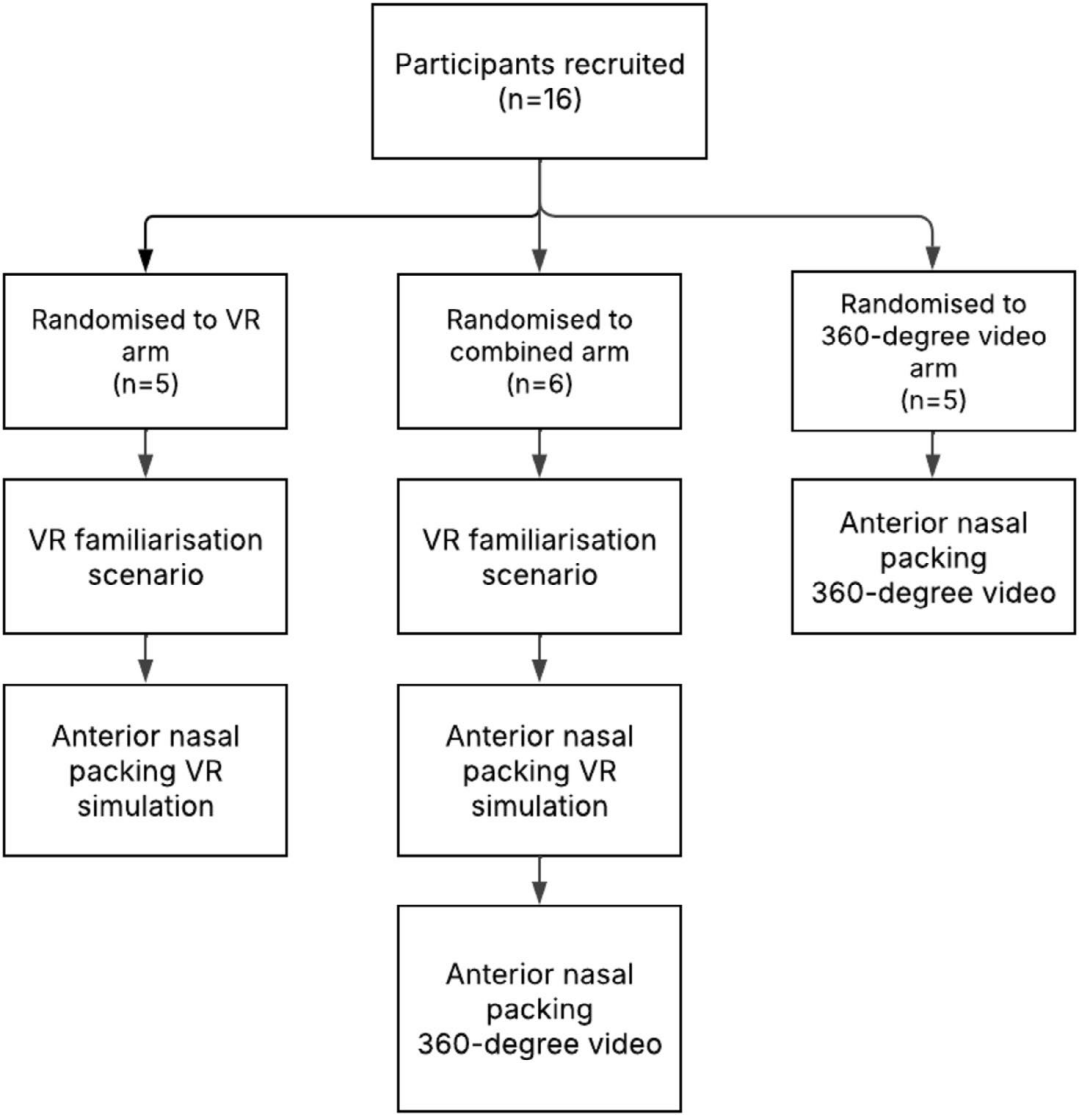
Study flow diagram illustrating activities undertaken by subjects in different arms of the study.

All participants were given a standardized introduction script. They accessed the ExR application and were shown how to access the relevant content. The sequence of activities undertaken by participants is shown in Figure 2. In the VR arm, participants were shown the controls using a standardized script and instructional poster. As a familiarization exercise, participants then undertook the subcutaneous injection simulation repeatedly until they felt subjectively comfortable with the controls. The author was present to answer questions regarding controls. The subjects then undertook the ANP simulation until they felt subjectively comfortable with both the content and the controls.

In the 360° video arm, participants watched the ANP video until they felt subjectively comfortable with both the content and the controls. In the combined arm, participants received introductions to controls and undertook the familiarization exercise as in the VR simulation arm. They undertook the ANP simulation repeatedly and then watched the 360° video until subjectively comfortable with the content and controls. You can access this live experience here.

### 2.6 Evaluation

Participants completed an online pre‐ and post‐session questionnaire. Both questionnaires were written by the author and derived using expert consensus (an ENT consultant and specialist trainee). The pre‐session questionnaire comprised demographic details, a 5‐question knowledge assessment, and subjective competence and confidence on a 5‐point Likert scale.

In the VR simulation and 360° video arms, post‐intervention questions on a 5‐point Likert scale evaluated subjective confidence and competence (2), content validity (5), face validity (4), systems usability scale (SUS) [21] (10), and global rating (6, plus 6 free‐text). They included the same knowledge assessment as in the pre‐session questionnaire (5). For the combined arm, the VR and 360° video questionnaires were merged such that there were separate face validity evaluations for the video and VR simulation and an additional free‐text question (see Supporting Information).

### 2.7 Statistical Analysis

Statistical analysis was performed using JASP (University of Amsterdam, Netherlands) version 0.18.3. Likert scale questions were assigned numerical values (1 = strongly disagree, 5 = strongly agree) unless otherwise stated. Due to non‐normality of the population, non‐parametric statistical tests were chosen. A Wilcoxon signed rank test was undertaken for each arm of the study to evaluate whether there was a statistically significant change in pre‐ and post‐intervention competence and confidence. Differences between pre‐ and post‐intervention confidence and competence were computed, and the values analyzed using a Kruskal‐Wallis test to determine if there was significance between arms of the study. Similarly, a Kruskal‐Wallis test was used to compare content validity between arms of the study. Pre‐ and post‐intervention participant knowledge was analyzed using a three‐way repeated measures ANOVA.

## 3 Results

Sixteen foundation year doctors were recruited over a period of 4 months to the VR (*n* = 5), 360° video (*n* = 5), and combined (*n* = 6) arms. Most participants were female (*n* = 9) and right‐handed (*n* = 14). Participants had a mean age of 25.4 years. Three participants had used VR previously, and their mean subjective gaming experience was medium (2.875). Most participants stated that they had previously learned about ANP (*n* = 10); but few had previously seen a clinician perform ANP (*n* = 4), and only two participants had performed ANP themselves previously.

### 3.1 Implementing the Interventions

The mean time taken to complete the VR simulation after the familiarization scenario was 6 min and 59.1 s (~7 min). The 360° video lasted 4 min and 26 s. All participants in the 360° video and combined arms played the video once. Participants in the VR and combined arms played the familiarization an average of 1.2 times, and the ANP simulation 1.1 times.

Three participants have been excluded from the above analysis: due to poor WiFi in the hospital (*n* = 2) and an intervention error (*n* = 1). The mean responses to the global ratings questions are shown in Table 1.

**TABLE 1:** Median scores for global rating statements from all participants (*n* = 16).

Global rating statement	Median
This system is relevant to my training	4
I would recommend this system to a colleague	4
I would like this system to be included as part of my usual training	4
I would be able to transfer skills learned through this system to my usual practice	4
After using this system, I am more interested in a career in ENT	3
I would like to use this system to learn other procedures	5

### 3.2 Confidence and Competence

Participants rated their mean pre‐intervention confidence in the steps of ANP as low (1.94, SD 1.09), which increased to high (4.06, SD 0.66) post‐intervention (***p* < 0.001**). Similarly, mean pre‐intervention subjective competence was low (1.88, SD 1.05), increasing to medium‐high (3.81, SD 0.73) post‐intervention (***p* < 0.001**). Whilst there were increases in confidence and competence in each arm, the increase in confidence of the combined arm was the only one to reach significance, which is demonstrated in Table 2. No significant differences were found between changes in confidence or competence between each arm in the study (*p*‐values 0.885 and 0.545 respectively).

**TABLE 2:** Pre‐ and post‐intervention confidence and competence medians (out of 5), their differences, and *p*‐values demonstrating whether this change was significant.

Arm	Number	Median pre‐intervention confidence	Median post‐intervention confidence	Difference in medians	*p*
VR	5	2	4	2	0.054
360	5	1	4	3	0.053
Combined	6	2	4	**2**	**0.034**
Whole cohort	16	1.5	4	**2.5**	**< 0.001**

		Median pre‐intervention competence	Median post‐intervention competence		
VR	5	2	3	1	0.053
360	5	1	4	3	0.053
Combined	6	2	4	2	0.054
Whole cohort	16	1.5	4	**2.5**	**< 0.001**

*Note:* Bold highlights indicate statistical significant findings.

### 3.3 Knowledge

Mean knowledge assessment scores are shown in Table 3. There was a statistically significant increase in knowledge scores across the whole study cohort and within each arm (***p* < 0.001**).

**TABLE 3:** Mean pre‐ and post‐session knowledge assessment scores (out of 5) for all arms, with *p*‐values demonstrating that all assessment score changes were significant.

Arm	Number	Mean pre‐intervention score (SD)		Mean post‐intervention score (SD)		Difference in means (SD)		*p*
VR	5	1.40	(1.1)	4.00	(0.0)	2.60	(1.0)	**0.007**
360	5	0.80	(1.1)	3.60	(0.9)	2.80	(1.0)	**0.005**
Combined	6	1.83	(1.3)	4.83	(0.4)	3.00	(1.2)	**0.002**
Whole cohort	16	1.38	(1.2)	4.19	(1.2)	2.81	(1.1)	**< 0.001**

*Note:* Bold items indicate statistical significance.

### 3.4 Usability

Participant ratings on the SUS were converted to scores out of 100 [21]. The average converted score was 70.94 (SD 17.76) for all technologies, with the VR, 360° and combined arms having scores of 62 (SD 19.6), 78 (SD 13.5), and 72.5 (SD 14.4) respectively. Given the standard deviations for mean SUS scores vary quite considerably, definitive conclusions about the acceptability of these technologies cannot be drawn.

### 3.5 Validity Assessment

Content validity for ANP was evaluated using five questions. Mean scores for each item is shown in Table 4. No significant differences in the mean content validity scores were found between study arms. All domains had a mean score greater than four apart from “this system taught the relevant anatomy for ANP,” as participants scored this lower in all three arms of the study. Given that each arm was given a mean score above 20 out of 25 (a benchmark used in similar studies [6, 16]), all technologies in this study achieved content validity.

**TABLE 4:** Median content validity results for all arms of the study.

	VR (*n* = 5)	360 (*n* = 5)	Combined (*n* = 6)	Whole cohort (*n* = 16)
This system taught the relevant anatomy for anterior nasal packing	3	3	4	3
This system taught the procedural steps for anterior nasal packing	5	5	5	5
This system taught the technique for anterior nasal packing	4	4	5	5
This system taught the equipment required for anterior nasal packing	5	4	5	5
This system is a useful training tool for anterior nasal packing	5	4	5	5
Mean total out of 25 (SD)	21.4 (3.4)	20.8 (2.3)	22.83 (1.8)	21.75 (2.7)

*Note:* All domains were scored out of 5 unless otherwise stated.

Face validity was assessed using separate questionnaires for the VR simulation and 360° video. Those in the combined arm undertook both questionnaires. As in Table 5, the mean total score for the 360° video was 13.45 (SD 2.0) out of a maximum of 15, with each domain having a mean score greater than four, therefore the 360° video achieves face validity. Conversely, the VR experience scored 15.55 (SD 2.8) out of 20, which equates to a mean score per domain of 3.89, indicating that the VR software did not meet face validity criteria. As shown in Table 5, the domain “the haptic feedback in this system was very realistic” had a mean score of 3.09 (SD 1.4) and was significantly lower than the mean score for all other domains assessing VR face validity (all > 4).

**TABLE 5:** Face validity results for the VR (*n* = 11) and 360° video software (*n* = 11).

Technology	Domain	Median score
VR	The appearance of the equipment was very realistic	4.00
	The haptic feedback in this system was very realistic	3.00
	The depth perception in this system was very realistic	4.00
	The quality of the graphics in this system were good	4.00
	Mean total out of 20 (SD)	15.55 (2.8)
360° video	The procedure could be observed well using this system	5.00
	The quality of the video graphics in this system were good	5.00
	The number of additional video views in this system was appropriate	5.00
	Mean total out of 15 (SD)	13.45 (2.0)

*Note:* All domains were scored out of five unless otherwise stated.

### 3.6 Free Text Responses

Thematic analysis of qualitative data from free text responses was performed by hand. Responses for each question were analyzed for keywords, which were then grouped into themes.

### 3.7 Engagement and Ease of Use

Six participants commented that the VR simulation and 360° video were fun to use. Participants enjoyed the novelty of these teaching modalities. One participant commented that they “have a history of gaming…to be able to apply it to medical training would be great.”

Participants from both the VR and 360° video arms found the software easy to use, stating that it was “intuitive,” “simple,” and “user‐friendly.” Participants enjoyed the interactivity in the VR and combined arms.

However, some participants commented that the controls within the VR simulation could have been improved. Four participants commented on the difficulty of performing twisting motions. Participants suggested that sensitivity of the controls could be increased, or the mechanical motion could be altered so the simulation felt less “clunky.” One participant stated they were “consumed in being able to control the VR” (rather than learning the procedure) but commented that it could be “helpful to learn the procedures if the person using it was very familiar with the controls.”

### 3.8 Immersion

Many participants felt the VR world was immersive, commenting that the quality of the graphics, haptic feedback, and background noises added to this. One participant stated that the software was “as close to practicing in person as you can be.” Participants commented that immersion, novelty, and ease of use enabled them to focus and engage on the learning activity.

Some of the participants were affected by WiFi issues, and the qualitative responses reflected this. One participant stated that “the poor internet made the quality of the video very poor,” and another commented on the video graphics being blurry which may have been due to network issues or headset adjustment. Three participants commented that they initially felt “disorientated” within the virtual environment, although two stated that they adjusted to this.

### 3.9 Educational Value

One VR participant stated there was “no pressure about getting things wrong because it was all virtual,” and another stated “doing the training a few times (3, 4) would make me very confident in the skill of nasal packing”. The 360° video was valued, with one participant stating they could “see from all angles.” Those in the combined arm valued using the technologies together, as they “reinforce the knowledge differently.”

### 3.10 Simulation Features

Several participants commented on the clarity of instructions, valuing that the procedure was broken into steps. One participant especially valued the time taken for consenting patients and hand sanitizing as it “forces reflection on content and makes it more likely for things to be replicated in real life.” Another participant commented that the software had a “good user interface.”

One participant stated that the patient model within the VR simulation was “unnerving at points” and suggested it should be more like a dummy or more realistic.

A problem with the current VR simulation was its centricity around right‐hand dominant participants. One left‐handed participant struggled with steps of the simulation due to this; that is, when the simulation requests the user to lift the patient's nasal tip using their non‐dominant hand—to complete this action the user must bring their left hand towards the patient's nasal tip.

### 3.11 Suggestions for Additional Content

Participants suggested a variety of procedures that they thought the software would be useful in teaching (shown in Table 6), noting that they thought the system would be more useful in teaching short and simple procedures with relatively few steps, rather than longer procedures such as operations.

**TABLE 6:** Participant suggestions for useful procedures.

Which procedures do you think this system would be useful to teach?
Basic medical skills: Catheterisation Cannulaton Intramuscular injections Nasogastric tube insertion Venepuncture Arterial blood gas Recording patient observations Clinical examinations Higher medical skills: Chest drains Ascitic drains Ascitic tap Lumbar puncture Joint aspirations Surgical skills: Wound packing Debridement Orthopedic procedures Ophthalmology procedures

One participant suggested that “some visuals about the anatomy” may be helpful at the pack insertion stage of the simulation. Another suggested several features that could be added to the simulation, including adding “replay” and “ask for help” buttons, and a “test mode,” where the user could run through the simulation without instructions to test themselves.

## 4 Discussion

This study comparing the effect of VR simulation and 360° video in their efficacy of teaching the procedural steps of ANP is the first of its kind. Despite being a small feasibility study, it demonstrated that both the VR simulation and 360° videos have their merits as educational tools and are especially valuable when used together.

The 360° video achieved face and content validity and performed above average on the SUS. As far as the authors are aware, 360° videos have not been used previously to teach ENT procedures, so these findings are particularly pertinent to the development of future educational resources. Participants valued the 360° videos combined with VR simulation as they felt that they reinforced the steps being taught in different ways. The combined arm demonstrated a significant increase in confidence post‐intervention. Additionally, the cohort as a whole had a significant increase in both confidence and competence post‐intervention.

Whilst educationally effective, the VR simulation alone required some usability updates which have been performed based on these results. The combined arm demonstrated a higher SUS scale than VR alone, suggesting a learning curve in using the system as seen throughout the literature [22, 23]. As VR becomes more embedded within education and participants have longer exposure to it, they are therefore likely to find such technology more user‐friendly.

Other similar studies have concluded that VR used to teach ENT has achieved face validity [15, 16], but unfortunately this was not true for the VR simulation used in this study. All domains apart from haptic feedback achieved face validity. Implementing robust haptic feedback increases the cost of the system and reduces the portability and accessibility, factors which need to be balanced carefully depending on the learning outcomes trying to be achieved. Participants found the VR simulation immersive and engaging, and participants stated that the immersion enabled them to focus on the learning activity. Given this VR simulation is far more immersive than current technologies used in ENT [2, 3, 4, 6], it could offer a superior learning experience to pre‐existing technologies, especially if usability and haptic fidelity are improved.

Participants liked the novelty of the technology, and those with gaming experience found it enjoyable to be able to apply their personal interests to education. The technology was fun, and notably, participants agreed that they would be able to transfer skills learned through video or VR to clinical practice. Participants would recommend the technology to a colleague and would like to use the technology to learn other procedures. A wide variety of other procedures to be developed in VR were suggested by the participants, with some already in existence on the ExR platform—for example, chest drain insertion [24] and lumbar puncture [25].

## 5 Limitations

This was a small‐scale feasibility study that relied on voluntary participation from busy Foundation year doctors at a single trust. To draw further statistically significant conclusions from the data, the number of participants should be increased; multiple centres should be enrolled. Given the positive findings from this novel study, there is a strong case for a further larger study.

There has been no long‐term follow‐up of educational impact. To ensure that educational impact is long‐lasting, future studies could explore knowledge retention over time.

## 6 Conclusion

This novel feasibility study has demonstrated promising findings. VR simulation and 360° videos are an engaging and enjoyable teaching modality that increase user confidence, competence, and knowledge. All technologies studied achieved content validity, and the 360° video also achieved face validity. The 360° video used alongside VR or individually was considered above average on the SUS. Participants would recommend these technologies to a colleague and were keen to learn other procedures using the technology. After adjustments to the VR technology, a further larger study is needed to examine long‐term knowledge retention and evaluate face validity. These results demonstrate that 360° videos alongside VR or alone are an effective teaching modality for ANP.

## Conflicts of Interest

Mr Jonathan Abbas acknowledges his position as Co‐Founder of ExR Solutions Limited (https://exr.education), is a shareholder in VREVo Ltd, and sits on the editorial advisory board of the Journal of Medical Extended Reality. Mr Nick Culley acknowledges his position as Co‐Founder of ExR Solutions Limited.

## Supporting information


**Data S1:** Supporting Information.
