# Supplementary material for: A Feasibility Study of Virtual Reality and 360° Video Training for Anterior Nasal Packing
Source: Laryngoscope Investig Otolaryngol. 2025 Sep 9;10(5):e70243. doi: 10.1002/lio2.70243 (PMC12418922; doi:10.1002/lio2.70243)
Supplement: Supplementary file 1 — Data S1: Supporting Information. [file LIO2-10-e70243-s001.docx]

**Supplementary materials**

Below are the full data collection tools used in the study

***Pre-session evaluation – all participants completed this***

*Section 1*

1.Participant ID

2.Date of birth

3.Gender

- Male
- Female
- Non-binary

4.Level of training

- FY1
- FY2

5.What is your handedness? i.e. Which hand is your dominant hand?

- Right-handedness
- Left-handedness

6.What is your intended career path?

- Medical speciality
- Surgical speciality
- General practice
- Not sure

7.Do you intend to pursue a career in ENT?

- Yes
- No
- Maybe

8.How certain are you that you will pursue your intended career path?

*1 is not at all certain, 3 is neutral, and 5 is very certain*

- 1
- 2
- 3
- 4
- 5

9.Have you used virtual reality in your medical training previously?

- Yes
- No
- Maybe

10.What has virtual reality been used to teach you previously?

- [Free text]

11.How would you rate your previous gaming experience?

*1 is no gaming experience at all, 3 is some gaming experience, and 5 is extensive gaming experience*

- 1
- 2
- 3
- 4
- 5

12.Have you learned about anterior nasal packing (otherwise known as the use of a Rapid Rhino/nasal tampon) previously?

- Yes
- No
- Maybe

13.Have you seen a clinician perform anterior nasal packing in clinical practice previously?

- Yes
- No
- Maybe

14.Have you performed anterior nasal packing in clinical practice previously?

- Yes
- No
- Maybe

15.How would you rate your confidence in knowing the procedural steps of anterior nasal packing?

*1 is not at all confident, 3 is neutral, and 5 is very confident*

- 1
- 2
- 3
- 4
- 5

16.How would you rate your competence in performing the procedural steps of anterior nasal packing?

*1 is not at all confident, 3 is neutral, and 5 is very confident*

- 1
- 2
- 3
- 4
- 5

*Section 2*

Pre-session knowledge assessment

Please choose the single best answer for the following 5 questions

17.At what angle should a rapid rhino be inserted into the nose?

- At 45 degrees throughout the whole insertion
- Parallel to the floor of the nose for the whole insertion
- Initially parallel to the floor of the nose and then at 45 degrees
- Initially at 45 degrees and then parallel to the floor

18.What demonstrates that the coating of the rapid rhino has been activated?

- The coating turns translucent
- The coating turns white
- The coating hardens
- The coating becomes warm

19.What activates the coating of the rapid rhino?

- Contact with the lining of the nose
- Submersion in sterile water
- Contact with the air
- Inflation of the rapid rhino balloon

20.Why is the rapid rhino balloon inflated prior to insertion?

- To activate the coating of the rapid rhino
- To make insertion easier
- To check the maximal volume of air that can be inserted
- To ensure the balloon has not been punctured

21.For how long should the rapid rhino remain in situ before the balloon can be deflated?

- 6 hours
- 12 hours
- 24 hours
- 48 hours

***Post-session evaluation***

*Section 1 –* ***all participants***

1.Participant ID

2.How would you rate your confidence in knowing the procedural steps of anterior nasal packing?

*1 is not at all confident, 3 is neutral, and 5 is very confident*

- 1
- 2
- 3
- 4
- 5

3.How would you rate your competence in performing the procedural steps of anterior nasal packing?

*1 is not at all confident, 3 is neutral, and 5 is very confident*

- 1
- 2
- 3
- 4
- 5

*Section 2*

Content survey – **all participants**

*All statements should be rated on a 1-5 scale, with 1 being strongly disagree, 3 being neutral, and 5 being strongly agree.*

To what extent do you agree with the following statements?

4.This system taught the relevant anatomy for anterior nasal packing

5.This system taught the procedural steps for anterior nasal packing

6.This system taught the technique for anterior nasal packing

7.This system taught the equipment required for anterior nasal packing

8.This system is a useful training tool for anterior nasal packing

*Section 3*

Post-session knowledge assessment – **all participants**

*Please choose the single best answer for the following 5 questions*

9.At what angle should a rapid rhino be inserted into the nose?

- At 45 degrees throughout the whole insertion
- Parallel to the floor of the nose for the whole insertion
- Initially parallel to the floor of the nose and then at 45 degrees
- Initially at 45 degrees and then parallel to the floor

10.What demonstrates that the coating of the rapid rhino has been activated?

- The coating turns translucent
- The coating turns white
- The coating hardens
- The coating becomes warm

11.What activates the coating of the rapid rhino?

- Contact with the lining of the nose
- Submersion in sterile water
- Contact with the air
- Inflation of the rapid rhino balloon

12.Why is the rapid rhino balloon inflated prior to insertion?

- To activate the coating of the rapid rhino
- To make insertion easier
- To check the maximal volume of air that can be inserted
- To ensure the balloon has not been punctured

13.For how long should the rapid rhino remain in situ before the balloon can be deflated?

- 6 hours
- 12 hours
- 24 hours
- 48 hours

*Section 4*

Virtual reality appearance survey – **VR and combined technologies arms**

The following 4 statements are about the appearance of the virtual reality simulation.
*All statements should be rated on a 1-5 scale, with 1 being strongly disagree, 3 being neutral, and 5 being strongly agree.*

To what extent do you agree with the following statements?

14.The appearance of the equipment was very realistic

15.The haptic feedback in this system was very realistic

*Haptic feedback refers to touch and vibrations from the controllers as you would expect when preforming procedures in real-life*

16.The depth perception in this system was very realistic

17.The quality of the graphics in this system were good

*Section 5*

360-degree video appearance survey – **360-degree video and combined technologies arms**

The following 4 statements are about the appearance of the 360-degree video.
*Statements should be rated on a 1-5 scale, with 1 being strongly disagree, 3 being neutral, and 5 being strongly agree.*

To what extent do you agree with the following statements?

18.The procedure could be observed well using this system

19.The quality of the video graphics in this system were good

20.The number of additional video views in this system was appropriate

21.In terms of additional video views in this system...

- I would have liked more views
- There was the right amount of views
- I would have liked fewer views

*Section 6*

User-friendliness survey – **all participants**

*All statements should be rated on a 1-5 scale, with 1 being strongly disagree, 3 being neutral, and 5 being strongly agree.*

To what extent do you agree with the following statements?

22.I would like to use this system frequently

23.I found this system unnecessarily complex

24.I thought this system was easy to use

25.I think that I would need the support of a technical person to be able to use this system

26.I found that the various functions of the system were well integrated

27.I thought there was too much inconsistency in the system

28.I would imagine that most people would learn to use this system very quickly

29.I found the system very cumbersome to use

30.I felt very confident using the system

31.I needed to learn a lot of things before I could get going with the system

*Section 7*

Global ratings – **all participants unless otherwise stated**

*All statements should be rated on a 1-5 scale, with 1 being strongly disagree, 3 being neutral, and 5 being strongly agree.*

To what extent do you agree with the following statements?

32.This system is relevant to my training

33.I would recommend this system to a colleague

34.I would like this system to be included as part of my usual training

35.I would be able to transfer skills learned through this system to my usual practice

36.After using this system, I am more interested in a career in ENT

37.I would like to use this system to learn other procedures

38.Which procedures do you think this system would be useful to teach?

- [Free text]

39.How did you feel whilst using the system?

- [Free text]

40.Why did you feel this way?

- [Free text]

41.What did you like about the system?

- [Free text]

42.What would you change about the system?

- [Free text]

43.How did you find the use of the virtual reality simulation alongside the 360-degree videos? – **combined technologies arm only**

- [Free text]

44.Do you have any other comments about the system?

- [Free text]
